# Intestinal fistula after magnets ingestion

**DOI:** 10.1590/S1679-45082013000200018

**Published:** 2013

**Authors:** Maurício Macedo, Manoel Carlos Prieto Velhote, Rafael Forti Maschietto, Renata Dejtiar Waksman

**Affiliations:** 1 Hospital Infantil Darcy Vargas, São Paulo, SP, Brazil; 2 Hospital das Clínicas, Faculdade de Medicina, Universidade de São Paulo, São Paulo, SP, Brazil; 3 Hospital Municipal Infantil Menino Jesus, São Paulo, SP, Brazil; 4 Hospital Israelita Albert Einstein, São Paulo, SP, Brazil

**Keywords:** Magnetics/adverse effects, Intestinal fistula/etiology, Foreign bodies, Play and playthings/injuries, Child, Case reports

## Abstract

Accidental ingestion of magnetic foreign bodies has become more common due to increased availability of objects and toys with magnetic elements. The majority of them traverse the gastrointestinal system spontaneously without complication. However, ingestion of multiple magnets may require surgical resolution. The case of an 18-month girl who developed an intestinal fistula after ingestion of two magnets is reported.

## RESUMO

A ingestão acidental de corpo estranho magnético tem sido mais observada, devido à disponibilidade cada vez maior de brinquedos e objetos com imãs. A maioria deles é eliminada pelo trato digestivo espontaneamente. Porém, a ingestão de duas ou mais peças podem desencadear situações de resolução cirúrgica. Relatamos aqui o caso de uma menina de 18 meses que desenvolveu fístula intestinal após a ingestão de 2 peças imantadas.

## INTRODUCTION

Ingestion of foreign bodies is a common complaint in pediatric emergency services. In the United States, the annual incidence is over 100 thousand patients, and more than 80% of the cases are observed in the pediatric population^(1)^. Most cases of foreign body ingestion occur in children aged between six months and three years. Among the artifacts more frequently ingested, coins, small toy parts, batteries and, less often, magnetic pieces, stand out. The object usually goes to the stomach, passes by the pyloric sphincter and ileocecal valve, and is naturally eliminated. In about 80% of cases the object is spontaneously eliminated; 20% require endoscopy, and complications occur in less than 1% and demand surgical procedures^(2)^.

Endoscopy is always indicated when the foreign body is in the esophagus or stomach and it is a long and sharp object, or multiple magnets, batteries, or if it stays in stomach, regardless of its size^(3)^. Surgery is reserved for complicated cases or when the object has not been eliminated. The most common complications are obstruction and perforation of the gastrointestinal tract.

Ingestion of magnetic pieces is still not frequent in Brazil; however it has distinct characteristics from other foreign bodies that should be known. In this report, we describe a complication related to ingestion of magnetic objects.

## CASE REPORT

**Figure 1:**
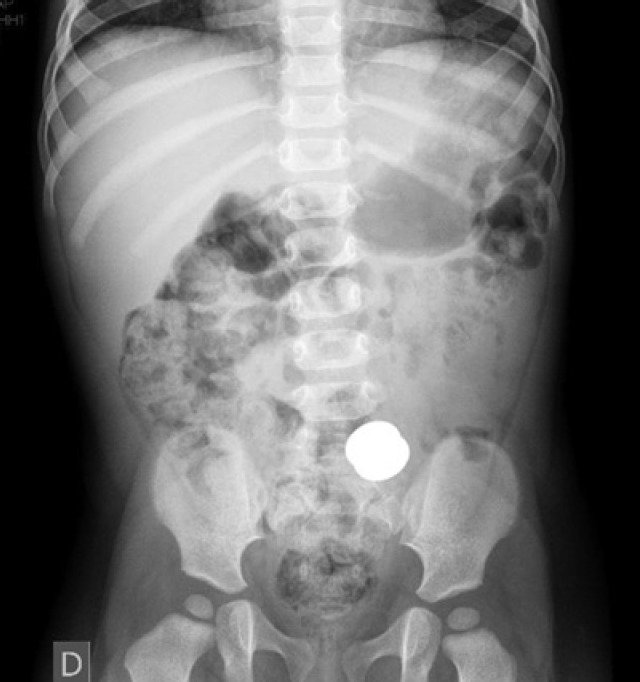
Simple abdominal radiograph showing a foreign body

**Figure 2:**
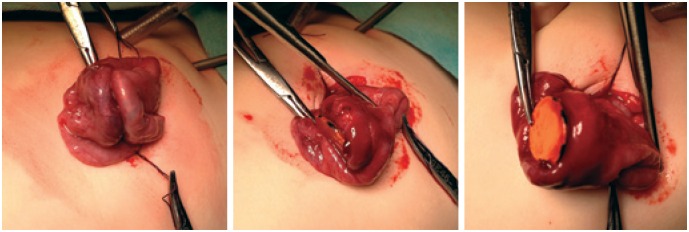
Intraoperative aspect showing one of magnets and the intestinal perforation

Seven days ago, an 18-month-old girl ingested two magnets, on the same day, which were used to attach photographs on a metallic surface. The patient was asymptomatic in this period, but the objects were not eliminated in stools. Upon physical examination, the abdomen was flacid, not painful on palpation and percussion, liver and spleen were not palpable, there were no tumors, and bowel sounds were normal. The radiograph taken on the day she ingested the pieces revealed artifacts in the abdominal cavity. Nevertheless, in radiological monitoring, the objects were in the same position in the mesogastrium. It was observed that the two magnetic pieces were together (Figure 1); thus, surgical treatment was chosen. Videolaparoscopy was initially indicated to locate the pieces. During the procedure it was noted that two intestinal loops were attached to and blocked by epyplon. When blockage was removed, the two magnets were seen in different bowel segments, and were 20cm away from each other; they were adhered together and there was a fistula between them (Figure 2). The bowel was exteriorized through the umbilicus, enterectomy was performed, the magnets removed and enteroenteral anastomosis was made. The patient progressed well and was discharged on the third postoperative day.

## DISCUSSION

Accidental ingestion of magnetic foreign bodies by children occur increasingly more often due to availability of objects and toys containing magnets^(4,5)^.

Ingestion of magnets and its complications are scarcely discussed in the literature. Most articles published are from Korea, Japan and China, where magnets are sold to treat muscle pain and improve circulation^(6,7)^. Magnets are also utilized as parts of toys and bijoux^(8)^.

The ingestion of a single magnet, small enough to go through the gastrointestinal tract, poses no additional risk. Likewise, the ingestion of two or more magnets presents no risk if the pieces are ingested simultaneously and if they adhere to each other. The complications occur when two or more pieces go through separately, in distinct segments, and one attracts the other when they are close. In this case, compression may lead to ischemia of the intestinal loop, resulting in complications, such as perforation and peritonitis^(8)^ or formation of fistula^(7,9)^, intestinal obstruction^(2,10)^ and small bowel volvulus^(11)^.

Once ingestion is diagnosed, if the objects are no longer in the stomach, patients must be submitted to strict observation, since the radiological differentiation of a single or more magnets can be subtle or even impossible to be determined.

Monitoring is made by radiographs when there are no signs of intestinal obstruction or perforation. The radiographic images should be taken after ingestion and repeated 48 to 72 hours later to assess progression of the object. If during monitoring the pieces are still in the same position, patients should be submitted to surgery.

Parents should be oriented to prevent such accidents. Healthcare professionals must be aware of the potential risks and possible complications of magnet ingestion, which should be treated differently from ingestion of other types of objects due to high associated morbidity.
